# Circulating Th22 cells, as well as Th17 cells, are elevated in patients with renal cell carcinoma

**DOI:** 10.7150/ijms.47384

**Published:** 2021-01-01

**Authors:** Zhiguo Peng, Yu Hu, Juchao Ren, Nengwang Yu, Zeyan Li, Zhonghua Xu

**Affiliations:** 1 Department of Urology, Qilu Hospital, Shandong University, 107 Wenhua West Road, Jinan 250012, PR China.; 2 Department of Medical Oncology, Qilu Hospital, Shandong University, 107 Wenhua West Road, Jinan 250012, PR China.

**Keywords:** renal cell carcinoma, Th22 cells, Th17 cells, interleukin-22

## Abstract

T-helper (Th) 22 cells serve an essential role in different types of tumors and autoimmune diseases. No research has been conducted to study the role of Th22 cells in the pathogenesis of renal cell carcinoma (RCC). We aimed to evaluate the prognostic value of circulating Th22, Th17, and Th1 cells in RCC patients. Thirty-two newly diagnosed RCC patients and thirty healthy controls were enlisted in the research. Their peripheral blood was collected, and the frequencies of circulating Th22, Th17, and Th1 cells were detected by flow cytometry. Plasma IL-22 concentrations were examined by an enzyme-linked immunosorbent assay (ELISA). Quantitative reverse transcription-polymerase chain reaction (RT-PCR) was used to identify the mRNA expression levels of aromatic hydrocarbon receptor (AHR) and RAR-associated orphan receptor C (RORC) in peripheral blood mononuclear cells (PBMC). Compared with the healthy control group, the frequency of circulating Th22 and Th17 cells and concentrations of plasma IL-22 were significantly increased in RCC patients. However, there was no significant difference in the frequency of Th1 cells. A positive correlation between Th22 cells and plasma IL-22 levels was found in RCC patients. Also, there was a significant positive correlation between Th22 and Th17 cells in RCC patients. An up-regulated expression of AHR and RORC transcription factors were also observed in RCC patients. As tumor stage and grade progressed, the frequencies of Th22 and Th17 cells and the level of plasma IL-22 significantly increased. Meanwhile, there was a positive correlation between Th22 and Th17 cells and RCC tumor stage or grade. Furthermore, patients with high Th22 or Th17 cells frequency displayed a decreased trend in survival rate. Our research indicated that the increased circulating Th22 and Th17 cells and plasma IL-22 may be involved in the pathogenesis of RCC and may be involved in the occurrence and development of tumors. Th22 cells, plasma IL-22, and Th17 cells may be promising new clinical biomarkers and may be used as cellular targets for RCC therapeutic intervention.

## Introduction

Renal cell carcinoma (RCC) represents 2-3% of all cancers worldwide and has a worldwide estimated incidence of approximately 300,000 cases and mortality of 129,000 deaths every year 1-3. Different factors, such as cigarette smoking, obesity, asbestos exposure, and regular use of analgesics, are thought to promote RCC development, but its etiology is still unknown 4-6. Although surgical nephrectomy is effective in treating RCC patients who have no evidence of metastases, controlling RCC that is at an advanced-stage remains a challenge due to the lack of reliable biomarkers 7, 8. Therefore, identifying reliable markers for early detection or use as a prognostic factor is essential.

Studies have shown that cellular immunity plays a vital role in the pathogenesis of RCC 9, 10. The CD4^+^ T cell subset reflects the immune function state and is critical in the maintenance of homeostasis and tumorigenesis 11. CD4^+^ T cells can differentiate into many active subsets, including T-helper (Th) 1, Th2, Th17, and Treg cells. Previous evidence has suggested that these Th subsets are associated with the pathogenesis and progression of some solid tumors 12-15. Th22 cells are a newly identified subset of CD4^+^ T cell that is characterized by the secretion of IL-22, but not IFN-γ or IL-17 16, 17. Th22 cells are similar to Th17 cells in the expression of chemokine (C-C motif) receptor 6 (CCR6) and CCR4. Compared with Th17 cells, Th22 cells express CCR10 but do not express CD161 18. Furthermore, Th22 cells differ from Th17 and Th1 cells in that they have low level expression of the Th1 and Th17 transcription factors T-bet and RORC, while AHR is considered to be the main transcription factor of the Th22 subset 19. Additionally, under conditions of coexisting IL-6 and tumor necrosis factor-α (TNF-α), naive CD4^+^ cells can differentiate into Th22 subtypes 17. Based on the above, Th22 cells represent a particular and terminally-differentiated Th subset 16.

The main effector cytokine of Th22 cells is IL-22. IL-22 exerts its biological effects by a heterodimeric transmembrane receptor complex composed of IL-22R1 and IL-10R2, as well as subsequent Janus kinase-signal transducers and activators of transcription (JAK-STAT) signaling pathways, consisting of STAT-3, Jak1, and Tyk2 20. Recent studies showed that Th22 cells and IL-22 might participate in the pathogenesis of some tumors, such as multiple myeloma 21, gastric cancer 22, hepatocellular carcinoma 23, and colorectal cancer 24. These studies suggest a possible role of Th22 cells in tumorigenesis, and it may represent a new type of tumor biomarker. As we all know, no data are available on the role of Th22 cells or IL-22 in the pathogenesis of RCC. Our study investigated the prevalence of Th22, Th17, and Th1 cells in peripheral blood of patients with RCC. The relevant quantitative mRNA expressions of transcription factors such as AHR and RORC were also investigated. Furthermore, we evaluated the associations of Th22 cells and IL-22 with different tumor stages or grades of RCC to identify their potential predictive and prognostic importance.

## Materials and methods

### Patients and controls

Thirty-two newly diagnosed RCC patients (20 males and 12 females; age range, 31-63 years; median age, 52 years) who had been scheduled for surgery in Qilu Hospital between June 2016 and October 2018 were enrolled in our research. RCC patients with other cancers or autoimmune diseases, including Crohn's disease, psoriasis, systemic lupus erythematosus (SLE), and rheumatoid arthritis (RA) were not included in our research. No patient had received anticancer therapy before the operation. Physical examination and computed tomography (CT)/ magnetic resonance imaging (MRI) was used for clinical staging. Tumors were classified and staged according to the American Joint Committee on Cancer's tumor-node-metastasis (TNM) classification system (Edition 8). Stages I-II and III-IV were regarded as early and advanced stages of the disease, respectively. The tumor grade was assigned according to the WHO/ISUP grading system. The critical clinical information of the RCC patients is given in Table 1. Thirty healthy controls (17 males and 13 females; age range, 23-49 years; median age, 28 years) were also enrolled in our research. This study was approved by the Medical Ethical Committee of Qilu Hospital of Shandong University. According to the Declaration of Helsinki, informed consent was obtained from all patients before participating in this research.

### Flow cytometric analysis of Th22, Th17, and Th1 cells

Measurements of intracellular cytokines by flow cytometry were used to reflect cytokine-producing cells. In short, heparinized peripheral whole blood (400µL) was incubated with an equal volume of Roswell park memorial Institute (RPMI)-1640 medium for 4h at 37°C in 5% CO_2_ under coexisting condition of 25 ng/mL of phorbol myristate acetate (PMA), 1.7 mg/mL of monensin, and 1mg/mL of ionomycin (all from Alexis Biochemicals, San Diego, CA, USA).

Monensin was used to inhibit intracellular transport mechanisms and caused cytokines to accumulate in the cells. PMA and ionomycin are pharmacological T cell activators that mimic the signals produced by the T cell receptor (TCR) complex, with the advantage of stimulating any antigen-specific T cells. When PMA activates cells, CD4^+^ has a tendency to be down modulated. For this reason, to define CD4^+^ T cells, we stained the cells with APC-Cy7-conjugated anti-CD3 and PE-Cy7-conjugated anti-CD8 monoclonal antibodies. After incubation for 20minutes at room temperature in the dark with the monoclonal antibodies, the cells were fixed, permeabilized, and stained with PE-conjugated anti-IL-17 monoclonal antibodies, FITC-conjugated anti-IFN-γ monoclonal antibodies, and APC-conjugated anti-IL-22 monoclonal antibodies. All antibodies were from eBioscience (San Diego, CA, USA). Isotype controls were used to ensure correct compensation and to confirm the specificity of the antibody. Stained cells were analyzed by flow cytometry using a FACS Calibur cytometer equipped with CellQuest software (BD Bioscience PharMingen, San Jose, CA, USA).

Th22, Th17, and Th1 cells were defined as CD3^+^CD8^-^IFN-γ^-^IL-17^-^IL-22^+^, CD3^+^CD8^-^IFN-γ^-^IL-17^+^IL-22^-^ cells and CD3^+^CD8^-^IFN-γ^+^, respectively.

### Quantitative real-time PCR analysis of transcription factors

Total RNA was isolated by TRIzol (Invitrogen, USA) according to the manufacturer's instructions. The Prime Script RT reagent kit (Perfect Real Time; Takara) was used for reverse transcription reactions in line with the manufacturer's instructions. Reverse transcription reaction was performed at 37°C for 15 minutes and then at 85°C for 5 seconds. Real-Time quantitative PCR was done in Roche Applied Science Light Cycler®480II Real-time PCR systems (Roche Applied Science) conforming to the manufacturer's recommendations. The Real-time PCR reaction included, in a final volume of 20μL, 1μL of cDNA, 10μLof 2 × SYBR Green Real-Time PCR Master Mix, and 1μL each of the forward and reverse primers. The primers of RORC, AHR, and the endogenous control β-actin were as follows: RORC: forward 5′-TTT TCC GAG GAT GAG ATTGC-3′ and reverse, 5′-CTT TCC ACA TGC TGG CTACA-3′; AHR: forward 5′-CAA ATC CTT CCA AGC GGC ATA-3′ and reverse, 5′-CGC TGA GCC TAA GAA CTG AAA G-3′; β-actin: forward 5′-CCT TCC TGG GCA TGG AGT CCT G-3′ and reverse, 5′-GGA GCA ATG ATC TTG ATC TTC-3′. PCR products were analyzed by melting curve analysis and agarose gel electrophoresis to confirm product size and ensure that no by-products were formed. The results of the targets were expressed relative to β-actin transcripts as the internal control. All experiments were repeated three times.

### IL-22 enzyme-linked immunosorbent assay

Peripheral blood samples were taken from a forearm vein into heparin-anticoagulant vacutainer tubes. Plasma was obtained from all subjects by centrifugation and stored at -80°C for determining the concentration of cytokine IL-22. Plasma IL-22 concentration was determined using the quantitative sandwich enzyme immunoassay technique in line with the manufacturer's instructions.

### Statistical analysis

The results are expressed as the median (range) or mean ± SEM (standard error of the mean). Comparisons between the two groups were analyzed by the Wilcoxon rank-sum test. In correlation analyses, the Spearman's correlation test was used for the non-normal distribution data, and the Pearson's correlation test was used for the normal distribution data. Patient overall survival was defined as the time interval between the date of surgery and the date of death or last follow-up. Deaths due to causes other than RCC were not included in the death record. The cumulative survival time was analyzed with the use of Kaplan-Meier method, and the log-rank test was used to compare group differences. All statistical tests were performed using GraphPad Prism 6.0 software (GraphPad, San Diego, CA, United States) and SPSS (version 17.0; SPSS, Inc., Chicago, IL, USA). *P*<0.05 was considered statistically significant.

## Results

### Increased percentage of circulating Th22 cells and plasma IL-22 levels in RCC patients

**Figure 1:**
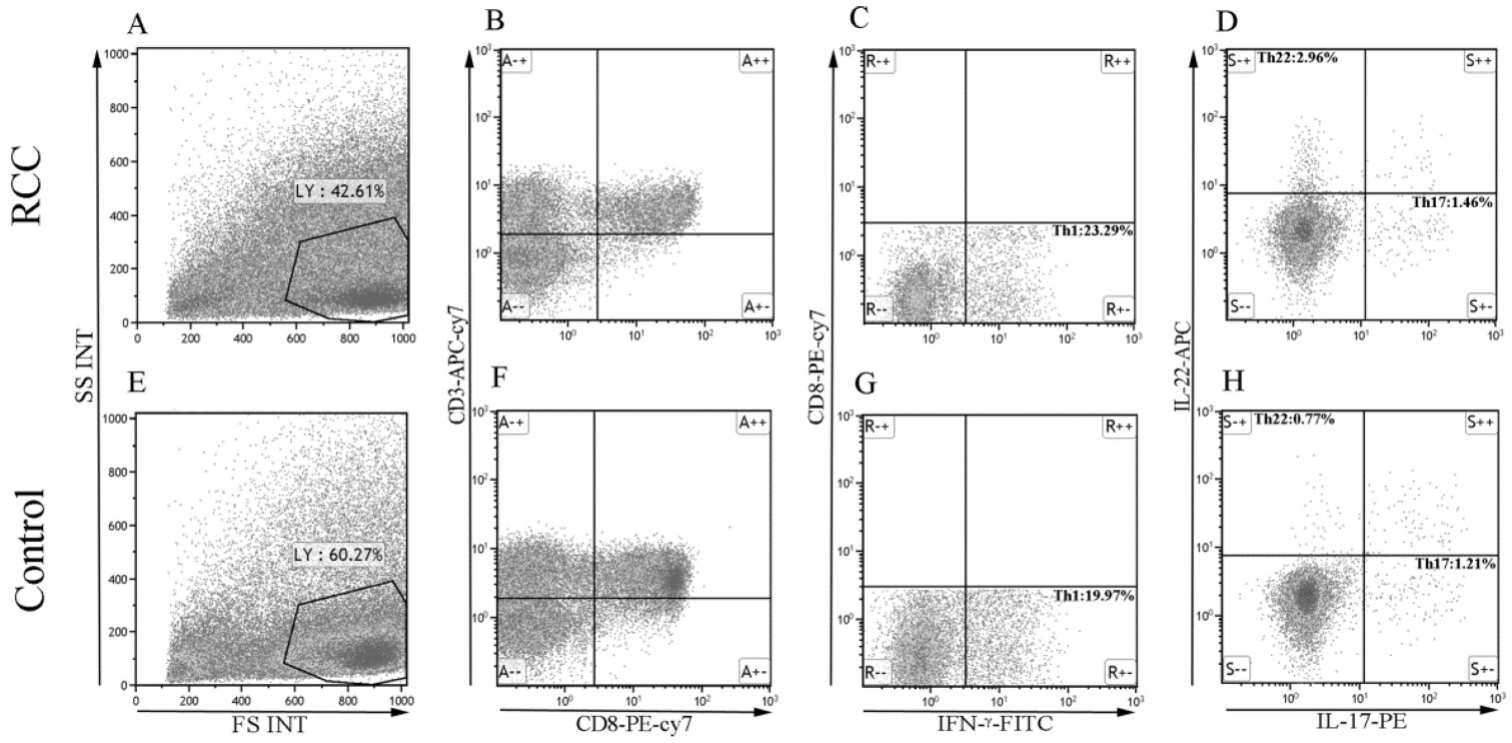
The percentage of Th22, Th17, and Th1 cells in representative RCC patients and healthy controls. (A, E) Circulating lymphocytes were gated by flow cytometry. (B, F) The percentage of circulating CD3^+^CD8^-^ lymphocytes was gated by flow cytometry. (C, G) The percentage of circulating CD3^+^CD8^-^IFN-γ^-^ or CD3^+^CD8^-^IFN-γ^+^ T cells was gated. (D, H) The percentage of Th22 (CD3^+^CD8^-^IFN-γ^-^IL-17^-^IL-22^+^) and Th17 cells (CD3^+^CD8^-^IFN-γ^-^IL-17^+^IL-22^-^) from RCC and healthy controls.

**Figure 2:**
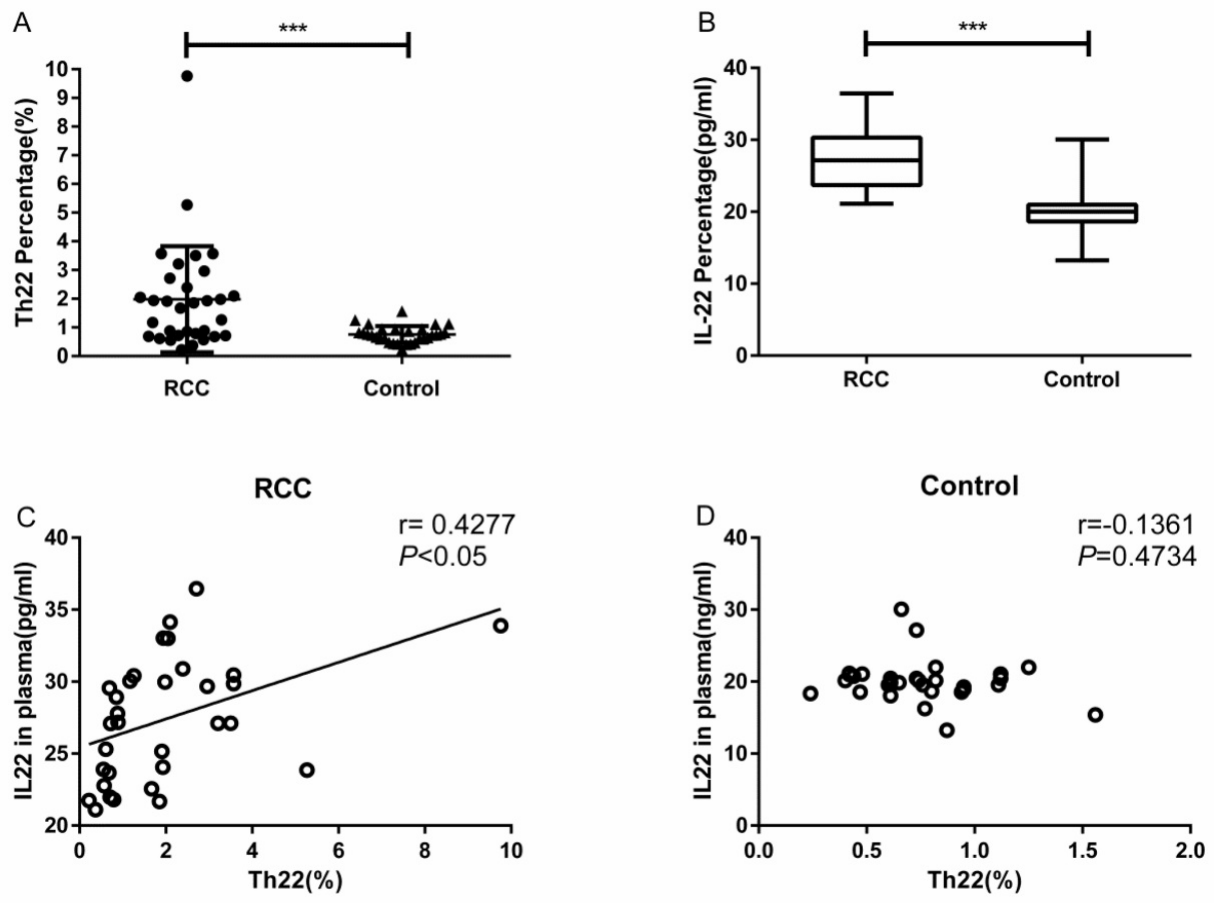
Th22 cells population and concentration of IL-22 in plasma from RCC patients and healthy controls. (A) Significantly elevated percentage of Th22 cells was found in RCC patients compared with healthy controls (1.97 ± 0.32% vs. 0.75 ± 0.05%, ***P<0.001). (B) IL-22 concentration was significantly increased in RCC patients compared with healthy controls (27.38±0.75 pg/ml vs.20.06±0.54 pg/ml ***P<0.001). (C) A positive correlation between Th22 cells and IL-22 was found in RCC patients (r=0.4277, *P<0.05). (D) The correlation was not found in healthy controls (r=-0.1361, P=0.4734).

Using multicolor flow cytometry, we examined the percentage of Th22 cells from the cytokine patterns after in vitro activation by PMA/ionomycin in short-term cultures. After gating on CD3^+^CD8^-^ cells, we identified Th22 (CD3^+^CD8^-^IFN-γ^-^IL-17^-^IL-22^+^), Th17 (CD3^+^CD8^-^IFN-γ^-^IL-17^+^IL-22^-^), and Th1 (CD3^+^CD8^-^IFN-γ^+^) cells. A representative dot plot of circulating Th22, Th17, and Th1 cells in typical RCC patients and healthy controls are presented in Fig. 1. Compared with the healthy controls, the percentage of circulating Th22 cells in total peripheral CD3^+^CD8^-^ lymphocytes of RCC patients was significantly increased (1.97 ± 0.32% vs. 0.75 ± 0.05%, ***P<0.001; Fig. 2A). A significant increase in the plasma IL-22 levels of RCC patients compared with healthy controls was also observed (27.38 ± 0.75 pg/mL vs. 20.06 ± 0.54 pg/mL ***P<0.001; Fig. 2B). Furthermore, a positive correlation between the percentage of Th22 cells and plasma IL-22 concentration was found in the RCC patients (r=0.4277, *P <0.05; Fig. 2C) while no correlation was found in the healthy controls (r=-0.1361, *P*=0.4734; Fig. 2D).

### Increased percentage of circulating Th17 cells in RCC patients

**Figure 3:**
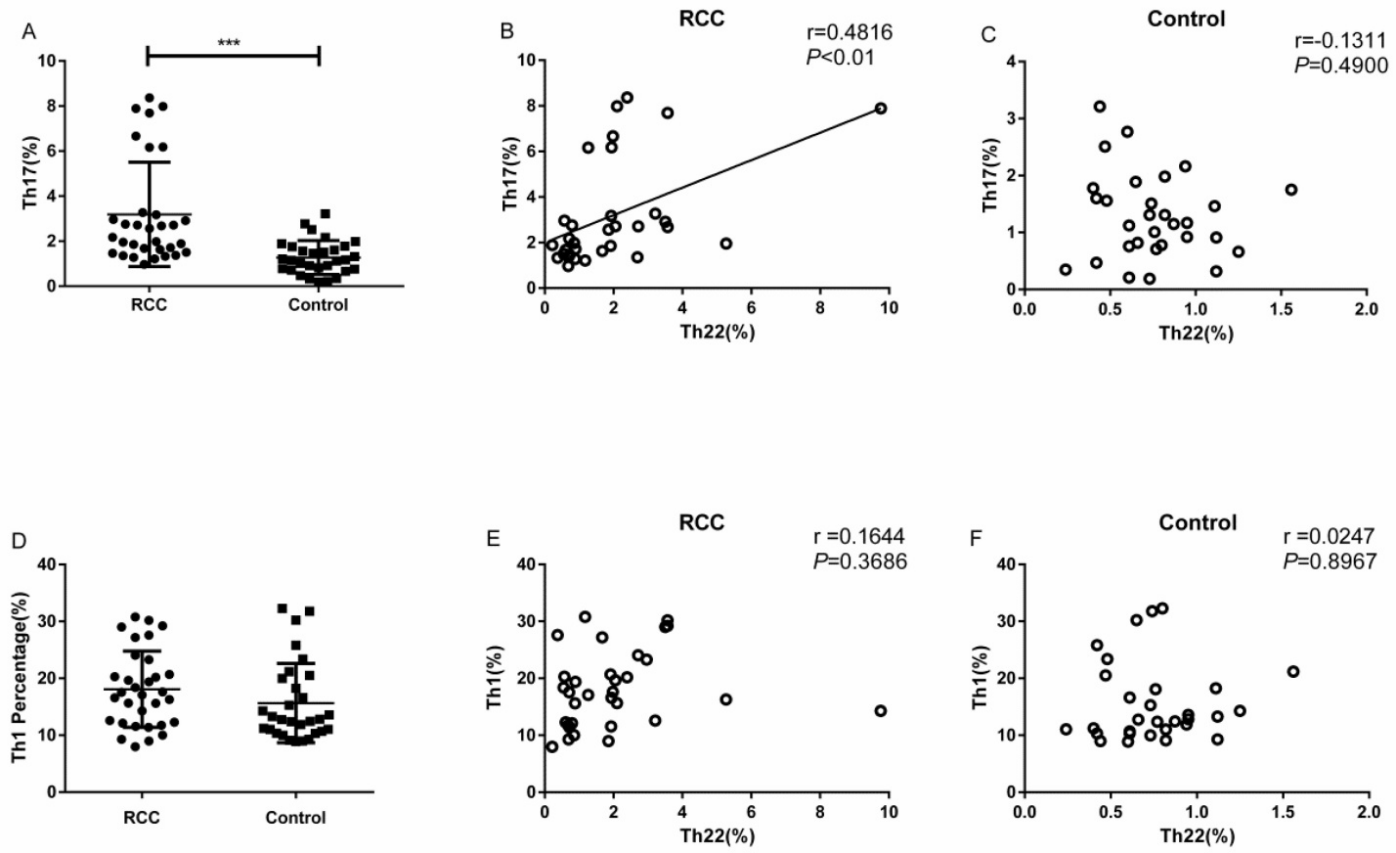
The percentage of Th17 cells with correlations between Th22 and Th17 or Th1 cells in RCC and healthy controls. (A) There was a significantly increased percentage of Th17 cells in RCC patients compared with healthy controls (3.18 ± 0.40% vs. 1.27 ± 0.13%, ***P<0.001). (B) A positive correlation between the percentage of Th22 and Th17 cells was found in RCC patients (r=0.4816, **P<0.01). (C) There was no correlation in the healthy controls (r=-0.1311, *P*=0.4900). (D) No significant difference was found in the percentage of circulating Th1 cells between RCC patients and healthy controls (18.08 ± 1.18% vs. 15.65 ± 1.27%,* P*=0.1676). (E, F) No significant correlation was found between Th22 and Th1 cells in RCC patients (r = 0.1644, *P* = 0.3686) or healthy controls (r = 0.0247, *P* =0.8967).

The percentage of Th17 cells in the RCC patients was significantly higher than the healthy controls (3.18 ± 0.40% vs. 1.27 ± 0.13%, ***P<0.001; Fig. 3A). Meanwhile, we found a positive correlation between Th22 and Th17 cells in RCC patients (r=0.4816, **P<0.01; Fig. 3B). Nevertheless, there was no significant correlation between Th22 cells and Th17 cells in the healthy controls (r=-0.1311, *P*=0.4900; Fig. 3C).

### Association between Th22 cells and Th1 subsets in RCC patients

There was no significant difference in the percentage of circulating Th1 cells between RCC patients and healthy controls (18.08 ± 1.18% vs. 15.65 ± 1.27%,* P*=0.1676; Fig. 3D). Furthermore, no significant correlation was found between Th1 and Th22 cells in RCC patients (r = 0.1644, *P* = 0.3686; Fig. 3E) or healthy controls (r = 0.0247, *P* =0.8967; Fig. 3F).

### Elevated AHR and RORC mRNA in RCC patients

**Figure 4:**
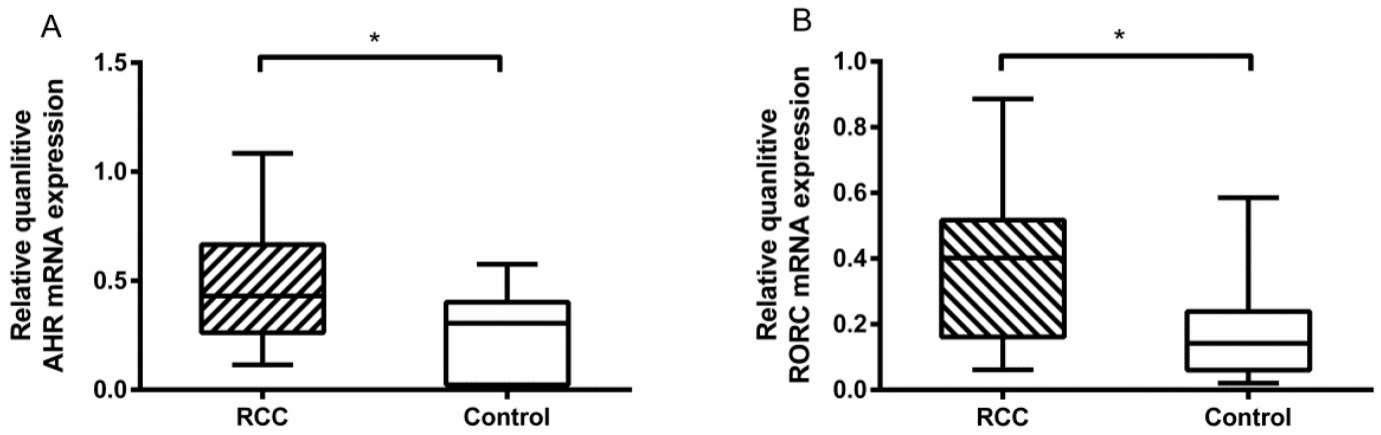
Quantitative RT-PCR for AHR and RORC expressions in RCC patients and healthy controls. (A) There was a higher measured value of AHR mRNA in RCC patients than healthy controls (0.47 ± 0.07% vs. 0.23 ± 0.05%, *P<0.05.) (B) There was a higher measured value of RORC mRNA in RCC patients than healthy controls (0.38 ± 0.06% vs. 0.18 ± 0.03%, *P<0.05).

We tested the related transcriptional factors of Th22 and Th17 cells by RT-PCR. The results showed that there was a higher level of AHR mRNA in the RCC patients than the healthy controls (0.47 ± 0.07% vs. 0.23 ± 0.05%, *P<0.05; Fig. 4A). Furthermore, the RORC mRNA level in the RCC patients was also higher than in the healthy controls (0.38 ± 0.06% vs. 0.18 ± 0.03%, *P<0.05; Fig. 4B). The results obtained confirmed the flow cytometry and ELISA data.

### Elevated proportions of Th22 and Th17 cells in different tumor stages

**Figure 5:**
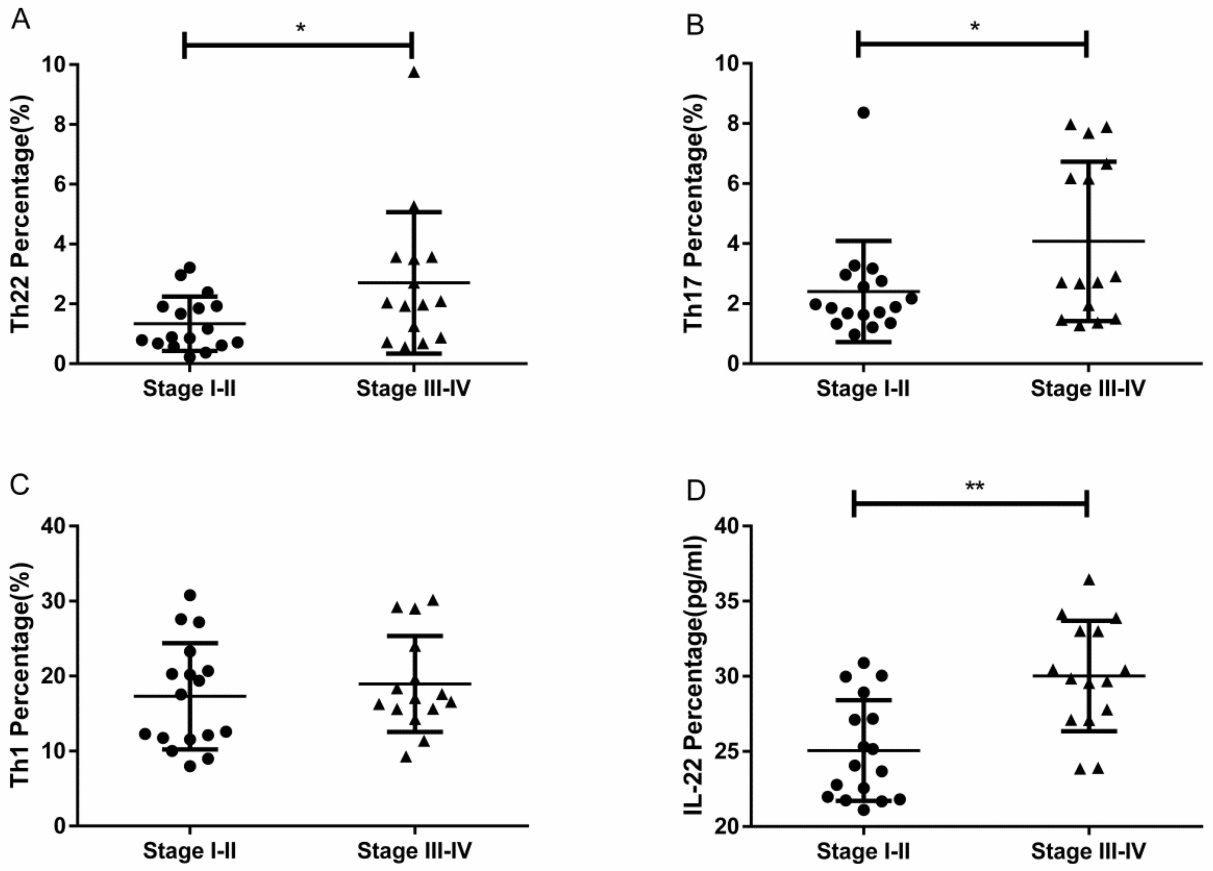
The percentage of circulating Th22, Th17, and Th1 cells and plasma IL-22 concentration in RCC patients with stage III-IV and stage I-II. (A) There was a significantly higher percentage of circulating Th22 cells in stage III-IV RCC patients than stage I-II patients (2.70 ± 0.61% vs. 1.34 ± 0.22%, *P<0.05). (B) There was also a significantly higher percentage of circulating Th17 cells in stage III-IV RCC patients than stage I-II patients (4.07 ± 0.68% vs. 2.40 ± 0.40%, *P<0.05). (C) There was no significant difference between the Th1 cell percentage and tumor stage in RCC patients (18.95 ± 1.65% vs. 17.31 ± 1.71%, *P*=0.4933). (D) There was a significantly higher concentration of IL-22 in stage III-IV RCC patients than stage I-II patients (30.02 ± 0.94 pg/ml vs.25.06 ± 0.81, **P<0.01).

RCC patients with stage III-IV had a significantly higher percentage of circulating Th22 cells than patients with stage I-II (2.70 ± 0.61% vs. 1.34 ± 0.22%, *P<0.05; Fig. 5A). Furthermore, the IL-22 concentration in stage III-IV patients was significantly elevated compared to patients in stage I-II (30.02 ± 0.94 pg/ml vs. 25.06 ± 0.81, **P<0.01; Fig. 5D). RCC patients with stage III-IV also had a significantly higher percentage of circulating Th17 cells than patients with stage I-II (4.07 ± 0.68% vs. 2.40 ± 0.40%, *P<0.05; Fig. 5B). No significant difference was found between the percentage of Th1 cells and tumor stage (18.95 ± 1.65% vs. 17.31 ± 1.71%, *P*=0.4933; Fig. 5C).

### Elevated proportions of Th22 and Th17 cells with different grades of RCC

**Figure 6:**
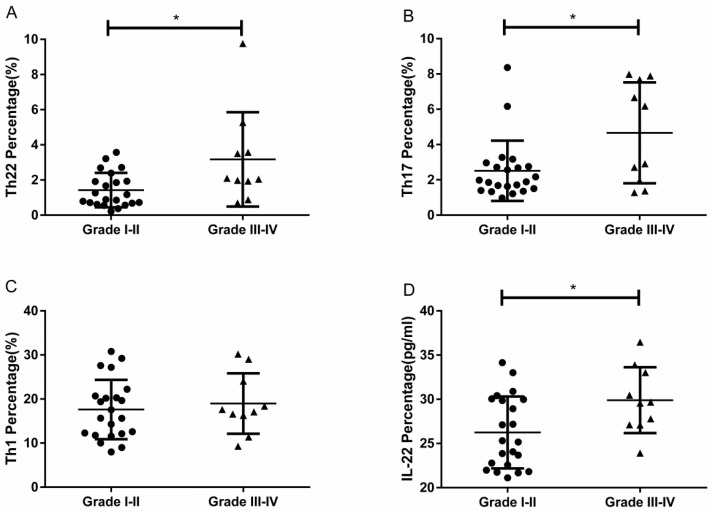
The percentage of circulating Th22, Th17, and Th1 cells and plasma IL-22 concentration in patients with different grades of RCC. (A) There was a significantly higher percentage of circulating Th22 cells in grade III-IV RCC patients than grade I-II patients (3.17 ± 0.84% vs. 1.42 ± 0.20%, *P<0.05). (B) There was also a significantly higher percentage of circulating Th17 cells in grade III-IV RCC patients than grade I-II patients (4.66 ± 0.90% vs. 2.51 ± 0.36%, *P<0.05). (C)There was no significant difference between the percentage of Th1 cells and tumor grade in RCC patients (18.98 ± 2.16% vs. 17.62 ± 1.43%, *P*=0.6004). (D) There was a significantly higher concentration of IL-22 in grade III-IV RCC patients than grade I-II patients (29.90 ± 1.17 pg/ml vs.26.24 ± 0.86, *P<0.05).

RCC patients with grade III-IV had a significantly higher percentage of circulating Th22 cells than patients with grade I-II (3.17 ± 0.84% vs. 1.42 ± 0.20%, *P<0.05; Fig. 6A). The IL-22 concentration in grade III-IV patients was significantly elevated compared to patients with grade I-II (29.90 ± 1.17 pg/ mL vs.26.24 ± 0.86, *P<0.05; Fig. 6D). RCC patients with grade III-IV also had a significantly higher percentage of circulating Th17 cells than patients with grade I-II (4.66 ± 0.90% vs. 2.51 ± 0.36%, *P<0.05; Fig. 6B). Nevertheless, there was no significant difference between the percentage of Th1 cells and tumor grade (18.98 ± 2.16% vs. 17.62 ± 1.43%, *P*=0.6004; Fig. 6C).

### The overall correlation of Th22, Th17 and Th1 cells with tumor stage and grade in RCC patients

**Figure 7:**
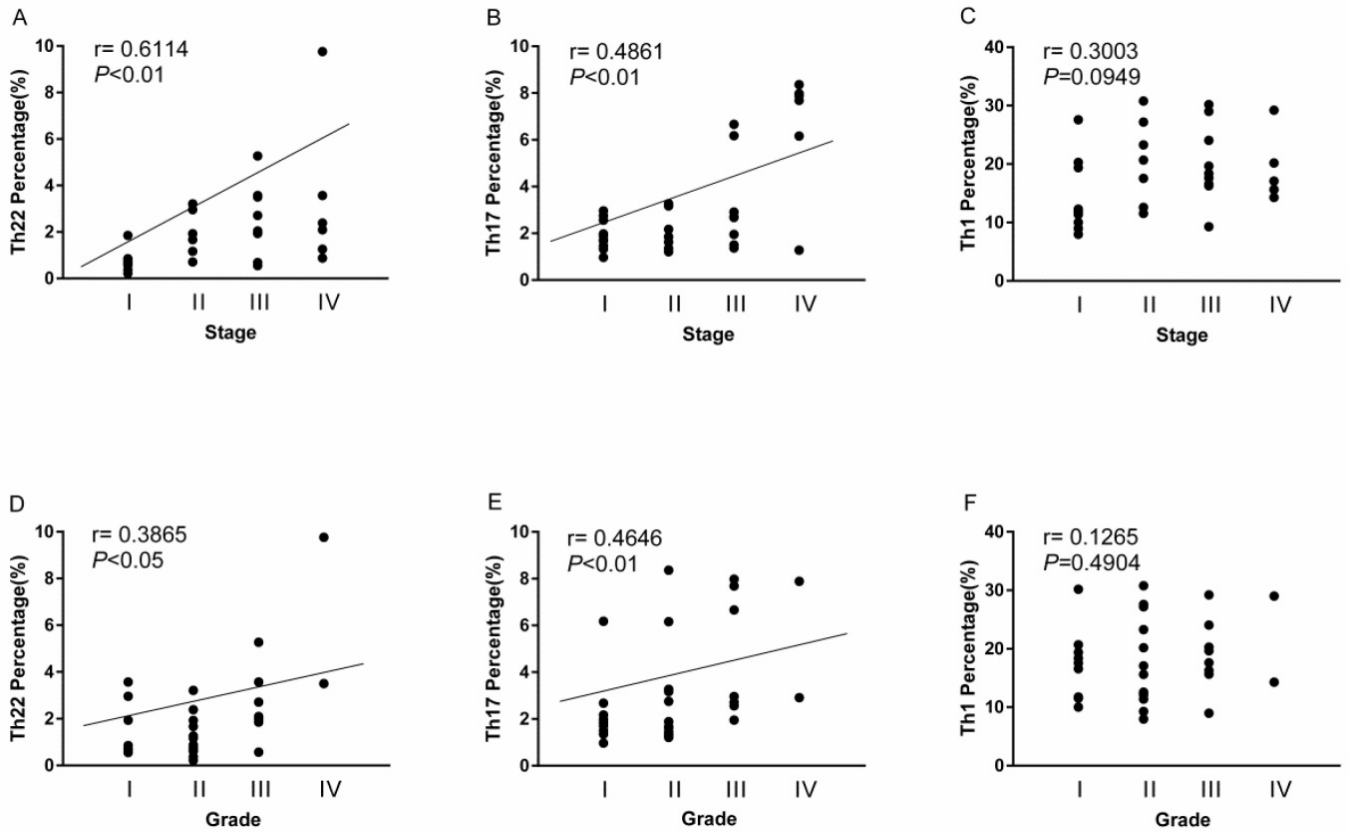
Association of circulating Th22, Th17, and Th1 cells with tumor stage and grade. (A, D) A positive correlation was found between Th22 cells and tumor stage (r=0.6114, **P<0.01) or grade (r=0.3865, *P<0.05) in RCC patients; (B, E) A positive correlation was also found between Th17 cells and tumor stage (r=0.4861, **P<0.01) or grade (r=0.4646, **P<0.01) in RCC patients; (C, F) There was no significant correlation between Th1 cells and tumor stage (r=0.3003, *P*=0.0949) or grade (r=0.1265, *P*=0.4904) in RCC patients.

A positive correlation was found between Th22 cells and tumor stage or grade in RCC patients (r=0.6114, **P<0.01; Fig. 7A; r=0.3865, *P<0.05; Fig. 7D). Meanwhile, there was also a positive correlation between Th17 cells and tumor stage or grade in RCC patients (r=0.4861, **P<0.01; Fig. 7B; r=0.4646, **P<0.01; Fig. 7E). Nevertheless, there was no significant correlation between Th1 cells and tumor stage or grade in RCC patients (r=0.3003, *P*=0.0949; Fig. 7C; r=0.1265, *P*=0.4904; Fig. 7F).

### The prognostic value of circulating Th22, Th17 and Th1 cells for the overall survival of RCC patients

**Figure 8:**
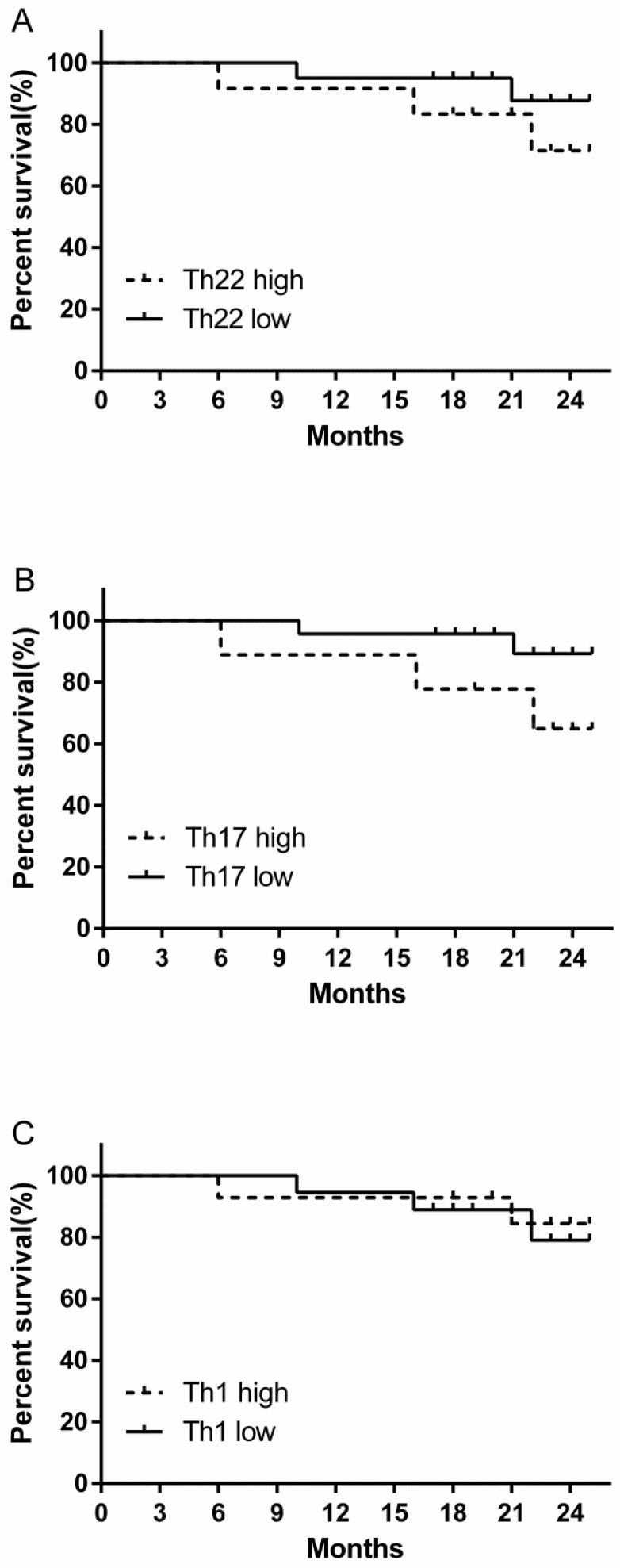
Kaplan-Meier curve for overall survival by median Th22, Th17 and Th1 cells in RCC patients. (A B) A decreased trend of survival rate was found in the patients with high Th22 or Th17 cell percentages compared with patients with low Th22 or Th17 cell level. (C) No similar trend of survival rate was found in patients between high versus low Th1 cell percentage level.

We evaluated the prognostic value of high and low levels of circulating Th22, Th17 and Th1 cells on the overall survival of RCC patients. There was a tendency for a decreased trend in the 24-month survival rate in the high percentage Th22 cell group compared to low percentage Th22 cell group, although this difference was not statistically significant (Fig. 8A). A similar survival rate trend was obtained when comparing patients with high versus low Th17 cell percentages (Fig. 8B). However, no similar trend of survival rate was found in patients between high versus low Th1 cell percentage level (Fig. 8C).

## Discussion

RCC was considered an immunologically sensitive cancer more than 30 years ago. Given that RCC tends to spread to distant sites, a reliable prognostic marker is required. It has been reported that some CD4^+^ T cells are essential for tumorigenesis and involved in different human cancers 25-27. Our research evaluated the probable role of preoperative circulating Th22 cells and the related Th subsets levels in RCC.

As expected, our study showed a higher proportion of circulating Th22 and Th17 cells in RCC patients compared with healthy controls. These results indicate that Th22 and Th17 cells may be related to T-cell-mediated immunity in RCC patients. Th22 cells have recently been defined as CD4^+^IFN-γ^-^IL-17^-^IL-22^+^ cells. It is a newly identified independent Th subset compared to the well-known Th1 and Th17 subsets 16. Th22 cells are thought to have an impact on certain tumors and autoimmune diseases. As we all know, little data is available on the role of Th22 cells in RCC. In our study, we found a significant increase in the frequency of Th22 cells in RCC patients compared to healthy controls, suggesting that Th22 cells may have a potential pathogenic role in RCC.

IL-22 is a member of the IL-10 cytokines family and is secreted mainly by activated Th22 cells 17. The role of IL-22 in cancer progression has been recognized in some epithelial cancers, such as breast and lung cancer. When IL-22 is released by immune cells, it can act on cancer cells to promote tumor growth, aggressiveness, and treatment resistance 28-30. Our study showed a significantly higher plasma IL-22 level in the RCC patients than the healthy controls. Furthermore, a significant correlation was found between the plasma IL-22 levels and Th22 cells in RCC patients, but no association in the healthy controls. A positive relationship between plasma IL-22 concentration and the frequency of Th22 cells in RCC may be explained by the fact that Th22 cells are a significant subset of IL-22 producing T cells, accounting for 37% to 63% of all IL-22-producing T cells 20.

Our current research also showed that Th22 cells increased with tumor stage and grade in RCC. Meanwhile, we observed a positive correlation between Th22 cells and tumor stage or grade. These data suggested that Th22 cells may be associated with tumor development and progression in RCC. We further studied AHR, the critical transcription factor directing Th22 lineage commitment, and found that the expression of AHR mRNA was increased in RCC patients. All of the above indicated that the Th22 subset was positively correlated with the occurrence and development of RCC and may promote tumor progression and affect the prognosis of RCC patients.

There has been controversy concerning the role of circulating Th17 cells in human tumor immunity 31. Our research showed that the frequency of Th17 cells in RCC patients was not only increased but also associated with both tumor stage and grade. We also found a significant positive correlation between Th22 and Th17 cells in patients with RCC. These results indicated that in the occurrence and development of RCC, abnormal differentiation of Th22 and Th17 cells may be induced in the same way.

RORC is the main transcription factor that plays a significant role in directing the Th17 lineage and modulates the polarization of Th22 cells 18. We observed a significant increase in RORC expression in RCC patients. Th17 and Th22 polarization require the transcription factors RORC and AHR. Also, Th22 differentiation requires the transcription factor AHR 32-34. Similarly, RORC and AHR are involved in IL-22 production 33, 34. Consequently, these associations may prime Th22 and Th17 cells and prompt a positive correlation between Th22 and Th17 cells in RCC patients.

Studies have suggested that Th1 cells play an anti-tumor role by mediating cellular immunity, activating CD8^+^ CTL cells, and promoting reproduction by secreting IL-2 and IFN-γ 35, 36. Therefore, we tested the frequencies of circulating Th1 cells to investigate its possible role in RCC patients. However, no significant difference was found in Th1 cell levels between RCC patients and healthy controls. Generally, Th1-mediated cellular immunity was thought to be related to early tumors with a shift toward other Th immune responses in advanced tumors 37. Onishi's study demonstrated that there was a change in the effective response from Th1 to Th2 with increasing stage of RCC 9. Although our research showed that the frequencies of Th1 cells were lower in RCC patients than in healthy donors, the difference was not significant.

Furthermore, Th1 cells showed no sign of correlation with Th22 cells in RCC patients. Also, no significant association was found between Th1 cells and tumor stage or WHO/ISUP grade in RCC. The specific function of Th1 cells in the tumor pathogenesis of RCC needs further investigation.

We also assessed the clinical value of Th22, Th17, and Th1 cells in the overall survival of RCC patients. Our study found that patients with high Th22 or Th17 cells frequency displayed decreased survival rates. Although this difference was not statistically significant, it may be clinically relevant in the long run.

In summary, our study demonstrated that the frequencies of circulating Th22 and Th17 cells were elevated in RCC patients compared with healthy controls. Furthermore, circulating Th22 and Th17 cells showed a significant association with advanced RCC tumor stage and high WHO/ISUP grade.

Given that there are few prognostic factors for RCC, circulating Th22 and Th17 cells might be useful clinical markers for assessing tumor diagnosis and progression of RCC. Further investigations on the functions of Th22 and Th17 cells in RCC patients may be conducive to designing novel therapeutic interventions in the future.

## Figures and Tables

**Table 1:** Clinical characteristics of the Study Subjects

Variable	RCC patients (n=32)	healthy controls (n=30)
age (median, range), y	52 (31-63)	28 (23-49)
Gender (male/female), n	20/12	17/13
TNM stage (I+II/III+IV), n	17/15	-
WHO/ISUP grade (I+II/III+IV), n	22/10	-
Pathology		-
Clear cell	29	-
Papillary	3	-
